# Digital Symptom Reporting for Treatment Readiness Before Daratumumab: A Blinded Prospective Study in Multiple Myeloma

**DOI:** 10.3390/hematolrep18030041

**Published:** 2026-06-15

**Authors:** Tine Rosenberg, Jannie Kirkegaard, Michael Tveden Gundesen, Anne Mette Ølholm, Karin Brochstedt Dieperink, Thomas Lund

**Affiliations:** 1 Department of Hematology, Odense University Hospital, Kløvervænget 10, 5000 Odense, Denmark; jannie.kirkegaard@rsyd.dk (J.K.); michael.tveden.gundesen@rsyd.dk (M.T.G.); thomas.lund2@rsyd.dk (T.L.); 2 Department of Clinical Research, University of Southern Denmark, Campusvej 55, 5230 Odense, Denmark; karin.dieperink@rsyd.dk; 3 Centre for Innovative Medical Technology, Odense University Hospital, Kløvervænget 30, 5000 Odense, Denmark; anne.mette.oelholm@rsyd.dk; 4 Research Unit of Oncology, Odense University Hospital, 5000 Odense, Denmark

**Keywords:** PRO, digital PRO, digital health, mobile application, multiple myeloma, daratumumab, clinical implementation, feasibility study

## Abstract

**Objectives:** For patients with multiple myeloma (MM), an increasing proportion of treatment is now delivered at home. While this shift offers convenience and supports continuity of care, it also demands new ways to ensure patient safety outside the hospital. Although patients receiving home-based treatment are generally clinically stable, each administration still requires a pre-treatment safety assessment, traditionally performed by telephone. We aimed to evaluate whether digital patient-reported outcomes (PROs) could replace these calls to determine treatment readiness. **Methods:** This feasibility study included 30 patients (median age 76 years, 60% male). Prior to each scheduled treatment, patients completed a digital symptom questionnaire. An algorithm stratified patients according to treatment readiness. In total, 233 questionnaires were distributed and 179 completed (completion rate 77%). Healthcare professionals were blinded to PRO data during the study, and PRO-based assessments were compared with standard nurse-led telephone evaluations after study completion. **Results:** Digital PRO data reliably identified patients ready for treatment. The algorithm achieved a positive predictive value of 100%, indicating concordance between PRO-based readiness classification and clinical decisions. The negative predictive value was 19%, reflecting that most patients reporting symptoms were ultimately eligible for treatment. Overall, nearly 80% of routine pre-treatment telephone calls could safely be omitted without compromising patient safety. **Conclusions:** This study suggests that digital PRO-based symptom reporting may support a reduction in routine pre-treatment telephone assessments for selected patients with MM receiving daratumumab. The approach showed potential for streamlining clinical workflows while maintaining patient safety, although confirmatory studies are needed before implementation.

## 1. Introduction

Multiple myeloma (MM) is an incurable hematologic malignancy characterized by uncontrolled proliferation of plasma cells in the bone marrow. Although newer therapies such as daratumumab have improved survival, patients often require continuous treatment and frequent monitoring, resulting in a substantial burden for both patients and healthcare systems.

To support patients in maintaining everyday life and to reduce pressure on healthcare services, home-based treatment models have increasingly been introduced in hematology care [1,2,3]. One example is subcutaneous (SC) daratumumab, which can be administered in patients’ homes or local healthcare clinics. However, these treatment models require assessment of treatment readiness reliably before each administration to ensure patient safety outside the hospital setting. Traditionally, this has been performed through nurse-led telephone consultations evaluating symptoms and general fitness for treatment. While clinically effective, these assessments are time-consuming and may be inconvenient for patients, highlighting the need for more efficient and patient-centered approaches.

Patient-reported outcomes (PROs) have increasingly been integrated into cancer care to support symptom monitoring and communication between patients and healthcare professionals. In solid cancers, digital PRO solutions have been shown to reduce unnecessary consultations and support remote clinical assessment without compromising patient safety [4,5,6,7,8].

However, evidence regarding the clinical use of PROs in hematologic malignancies remains limited, despite patients with hematologic cancers often following complex, long-term treatment trajectories involving frequent symptom monitoring and healthcare contact.

In 2022, we demonstrated that a digital PRO-based approach could support the replacement of standardized pre-treatment telephone assessments in patients receiving bortezomib [9]. The solution was integrated directly into the electronic health record system and has since been implemented in routine clinical practice.

Building on these experiences, the present study aimed to evaluate whether digital PRO-based symptom reporting could support treatment-readiness assessment before daratumumab in patients with MM. In addition, we explored the feasibility of integrating this approach into routine clinical practice.

## 2. Materials and Methods

### 2.1. Design

This was a prospective, non-randomized feasibility study with a parallel mixed-methods design. The intervention consisted of a digital PRO-based treatment readiness assessment. Due to the exploratory feasibility design, no formal sample size calculation was performed, as the study was not designed to assess non-inferiority or definitive safety outcomes. During the study period, nurses were blinded to PRO responses to ensure that treatment readiness evaluations were based solely on standard clinical procedures. Quantitative data were reported in accordance with the EQUATOR STROBE guidelines for observational studies [10], and qualitative data were reported following the COREQ (Consolidated Criteria for Reporting Qualitative Research) checklist [11].

### 2.2. Setting and Study Population

The study was conducted at a single tertiary hematology center as part of a clinical trial evaluating the feasibility of SC daratumumab being partly administered by primary care nurses either in patients’ homes or in local healthcare clinics, depending on patient preference and local municipal arrangements for treatment administration [3]. Participation in digital symptom reporting and treatment relocation could not be chosen independently. Patients eligible for inclusion were adults (>18 years), had a diagnosis of relapsed MM, were in possession of a smartphone or computer, and were planning to start or were already in treatment with daratumumab. Patients received daratumumab either as monotherapy or in combination with standard anti-myeloma regimens according to routine clinical practice, as previously described. Treatment schedules followed routine clinical practice as previously described [3].

Prior to each treatment administered outside the hospital, patients were contacted by a specialized hematology nurse to ensure they were clinically fit for treatment. Some daratumumab administrations remained hospital-based according to routine clinical practice, particularly during treatment initiation and clinically indicated visits. Prior to these hospital-based administrations, patients underwent standard clinical assessment by hematology staff. Further details on the treatment schedule and procedures have been described previously [3]. Moreover, patients completed a digital questionnaire prior to each scheduled treatment addressing the most important side effects of daratumumab. During the study period, nurses and physicians independently assessed treatment readiness according to usual clinical practice while remaining blinded to the PRO responses. Concordance between PRO-based triage and routine clinical assessments was evaluated retrospectively. Most patients received their first study treatment (C1D1) on the day of inclusion and therefore did not fill in a questionnaire prior to that treatment.

### 2.3. Data Collection

**Figure 1:**
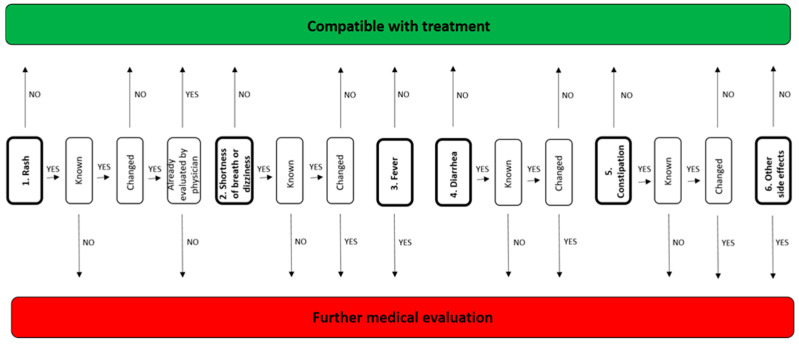
Algorithm stratifying patients according to treatment readiness based on digital symptom reporting. Bold and numbered boxes indicate mandatory questions. Patients were also able to report additional symptoms through a free-text “other side effects” option. Final triage outcomes were determined by the combination and development of reported symptoms rather than individual symptoms alone; therefore, some symptoms could result in either treatment readiness or further clinical evaluation depending on the overall response pattern.

Quantitative data were primarily obtained directly from patients through a secure, regionally provided digital health platform, as previously described [9]. Based on clinical experience, we developed a PRO measure addressing the most relevant side effects of daratumumab, including rash, shortness of breath, dizziness, fever, diarrhea, and constipation. Patients could further report additional symptoms through a free-text field. Based on patient responses, an algorithm categorized patients as either treatment-ready (green coded) or requiring further clinical evaluation (red coded) (Figure 1). When patients left the study, they were further provided a custom-built questionnaire evaluating self-reporting of side effects. Patients were contacted in case of missing treatment-readiness questionnaire responses. Other quantitative data were collected from the patients’ medical records.

Qualitative data were obtained from individual, semi-structured interviews with patients. We planned to include 10 consecutive patients initially, with up to three additional participants added until data saturation was reached [12,13]. A trained nurse not further involved in patient treatment conducted the interviews. The interviews were conducted by phone at a time point chosen by the patient and lasted 10–15 min. A semi-structured interview guide based on previous experiences [9] and existing literature was used. All interviews were recorded and transcribed verbatim.

### 2.4. Data Analysis

Quantitative data were summarized using descriptive statistics and presented as numbers, percentages, medians, and ranges. Due to the exploratory feasibility design and limited sample size, no formal statistical comparisons between groups were performed. Agreement between PRO-based triage and routine clinical assessment was evaluated by calculating positive and negative predictive values using the standard clinical assessment as the comparator. Data were analyzed using Stata BE 17.

Qualitative data were analyzed applying a hermeneutic approach. Systematic text condensation was performed according to Malterud, and transcripts were reviewed throughout the coding process to ensure that themes reflected the original data and their context [14]. Data analysis was performed using the electronic program NVivo Version 13.

## 3. Results

### 3.1. Study Population

Between March 2022 and June 2023, 54 patients were invited to participate in the clinical trial evaluating home-based daratumumab administration and digital symptom reporting. Of these, 30 patients (18 males and 12 females) with a median age of 76 years (range 61–87) were included (see Table 1). The main reason for declining was reluctance toward treatment relocation to primary care or home-based administration (*n* = 21), while three patients declined due to not owning a smartphone. Further details on declining patients have been described previously [3].

In the individual patient interviews, data saturation was reached when consecutive interviews no longer contributed new themes or perspectives relevant to the study aim [12,13]. This occurred after the inclusion of 19 consecutive patients.

### 3.2. Safety and Feasibility

In total, 269 treatments were administered in the study. Of these, 30 treatments occurred on the day of inclusion without prior side effect reporting through the digital platform. Nine questionnaires were not sent, while treatment postponement resulted in three additional questionnaires. As a result, 233 questionnaires were available for completion. Of these, 179 were completed, yielding a completion rate of 77%.

Reasons for non-completion included patients forgetting to respond and being unreachable (*n* = 4), and repeated difficulties completing digital symptom reporting using the smartphone-based platform (*n* = 50). Six patients accounted for 41 of the 54 non-completed questionnaires.

**Figure 2:**
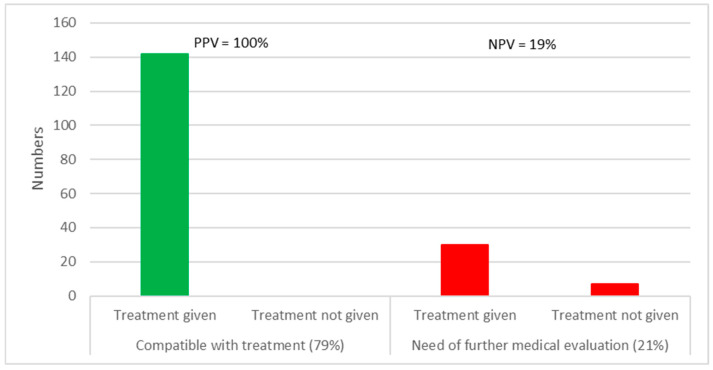
Self-reporting of side effects prior to treatment. Green: no side effects or no worsening in known side effects. Red: new or worsened side effects with a need for further medical evaluation prior to a final decision on whether to have the planned treatment. In 79% of cases, the algorithm correctly identified patients ready for treatment without requiring further evaluation and could therefore replace standard clinical assessments.

Of the 179 completed questionnaires, 142 had green-coded responses with the algorithm recommending treatment, and 37 were flagged in red. In all cases, green codes corresponded with treatment being administered after clinical evaluation. The algorithm demonstrated a positive predictive value of 100%, suggesting that digital PRO data may support a reduction in routine clinical evaluations in 79% of cases. With a negative predictive value of 19%, 30 of 37 treatments linked to a red-coded questionnaire response were administered (Figure 2). Reasons for red-coded responses are provided in Table 2.

### 3.3. Evaluation

Of the 30 patients included in the study, 25 (83%) filled out the evaluation form when they left the study. Reasons for non-response included early study withdrawal and limited experience with the app (*n* = 3) and the patient forgetting to respond (*n* = 2).

**Figure 3:**
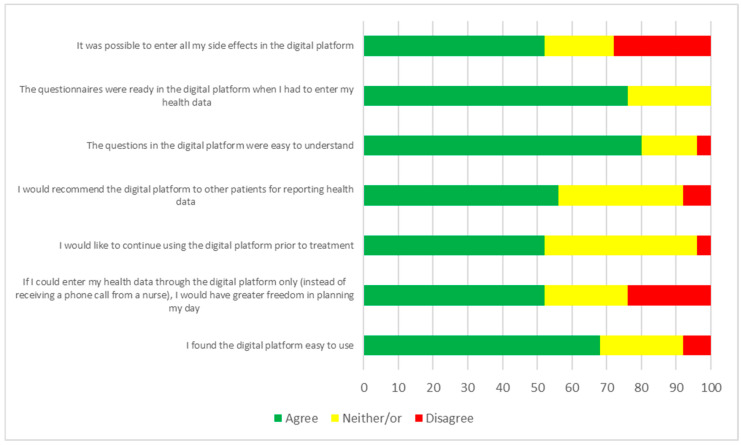
Patient evaluation of self-reporting of side effects using the digital platform. Numbers in percentages.

Of 25 respondents, 17 (68%) found the digital platform easy to use. Fifty-two percent (*n* = 13) preferred registering their side effects themselves, and 56% (*n* = 14) would recommend the platform to other patients (Figure 3).

Most patients found the platform easy to understand (80% agreed and 16% neither agreed nor disagreed) and experienced the questionnaires as accessible and available when needed (76% agreed and 24% neither agreed nor disagreed). This was also reflected in the interviews. One patient stated:

*“It shows up in the app, saying there are two messages from the hospital. Then I have to respond to them. I think that’s fine.”*

*(Male, 70 years)*

However, not all patients perceived the solution positively. In addition to the three patients declining participation because they did not own a smartphone, other patients reported challenges related to the platform during the interviews. One patient said:

*“It’s too complicated. It’s the app—we can’t get into our app because we can’t remember the password.”*

*(Male, 84 years)*

Another patient expressed that it was not the technology, but rather the lack of contact with the healthcare staff, that made her skeptical of the solution. She said:

*“I have enjoyed my conversations with the doctors, and now I’m losing that… I might just need someone to talk to.”*

*(Female, 68 years)*

## 4. Discussion

This study suggests that PRO data collected through a digital platform may help identify patients with MM ready for their next dose of daratumumab. With a positive predictive value of 100%, the algorithm suggested the potential to reduce routine telephone evaluations in nearly 80% of cases, thereby potentially reducing clinical workload. While the low negative predictive value (19%) indicates that patients reporting side effects would still require follow-up contact, the algorithm was intentionally designed conservatively, such that any reported symptom triggered additional clinical assessment rather than automatic treatment approval. In this context, the findings suggest that PRO-based triage may offer a pragmatic way to direct clinical attention toward cases where treatment eligibility is uncertain [15].

However, the findings should be interpreted within the context of a small exploratory single-center feasibility study; confirmatory studies are needed before broader implementation can be recommended. Furthermore, PRO-based triage was compared with routine nurse-led clinical assessment rather than an independent gold standard such as physician examination or prospective adverse-event outcomes. Accordingly, the results should be interpreted as reflecting concordance with existing clinical workflow rather than definitive validation of clinical safety or treatment-readiness assessment.

Several studies in solid tumor oncology have demonstrated that digital PRO monitoring can support remote symptom assessment and reduce unnecessary consultations without compromising patient safety [4,5,6,7,16,17]. As treatment pathways increasingly move beyond the hospital setting, efficient and safe approaches to remote symptom monitoring are becoming increasingly important. Existing digital PRO systems in cancer care primarily focus on symptom monitoring, patient education, or supporting communication between patients and healthcare professionals [18,19,20,21], while only a few have been developed as stand-alone tools for treatment-readiness assessment [7,22]. Moreover, evidence regarding routine PRO use in hematologic malignancies remains limited [8,9,22,23]. In this context, the present study adds to the emerging field by supporting the feasibility of digital symptom reporting for treatment-readiness assessment in routine hematology care. The present study builds on earlier work evaluating digital PRO-based pre-treatment assessments in patients receiving bortezomib, where a similar approach showed potential to replace routine telephone consultations in selected patients [9]. However, inclusion and response rates were lower in the present study (55% vs. 91% and 77% vs. 98%, respectively). This likely reflects that the current study was embedded within a broader clinical trial involving both digital symptom reporting and relocation of daratumumab treatment from hospital to patients’ homes or local health clinics [3]. Because participation in digital symptom reporting and treatment relocation could not be chosen independently, the observed inclusion, response, and satisfaction rates cannot be interpreted as reflecting acceptance of the digital PRO intervention alone.

The combined introduction of home-based care and digital symptom assessment may also have influenced patient satisfaction levels. One patient expressed: “I have enjoyed my conversations with the doctors, and now I’m losing that… I might just need someone to talk to.” Whether this reflected the loss of telephone contact, relocation of treatment, or both remains unclear and should be considered when interpreting the feasibility and acceptability findings.

The findings additionally highlight important challenges related to digital literacy in older hematology populations. Although only three patients declined participation because they did not own a smartphone, several enrolled patients experienced repeated difficulties using the digital platform, accounting for the majority of non-completed questionnaires. Thus, while digital PRO-based workflows may be feasible for many patients, some may require caregiver support, additional training, or alternative non-digital pathways. Accordingly, digital symptom reporting should currently be viewed as a complementary approach rather than a universal replacement for conventional clinical assessment.

In designing the PRO component, we deliberately developed a tailored screening tool rather than adopting a validated questionnaire. The aim was not longitudinal symptom measurement or comparison of symptom burden across patients, but rapid binary treatment-readiness assessment integrated into a specific clinical workflow. Therefore, concise yes/no questions were considered more clinically relevant than detailed symptom grading. A limitation of this approach is that the questionnaire was not formally psychometrically validated, and no reliability testing was performed prior to implementation. While this was considered acceptable within the exploratory feasibility framework of the present study, future studies should evaluate the reliability, usability, and clinical performance of the tool more systematically before broader implementation.

A key strength of the solution was its direct integration into the electronic patient record system, allowing both nurses and physicians to access patient-reported data without switching platforms. The red/green algorithm provided a simple visual overview of treatment readiness and need for follow-up assessment, thereby supporting workflow efficiency and clinical prioritization. The solution has since been fully implemented in routine clinical practice, supporting its feasibility and applicability in everyday patient management.

## 5. Conclusions

This feasibility study suggests that digital PRO-based triage may support the replacement of some routine pre-treatment telephone assessments for selected patients with multiple myeloma receiving daratumumab. Although the findings indicate potential for reducing clinical workload while maintaining patient safety, confirmatory studies are needed to validate the approach and clarify its applicability across broader patient populations.

Importantly, the intervention was developed with long-term feasibility in mind: concise, binary items tailored to the clinical workflow, direct integration with the electronic health record, and intuitive visualization for healthcare staff. These design features facilitated successful implementation, and the solution is now embedded in daily clinical practice. Our findings support broader efforts to integrate digital PRO into hematology, a field where evidence remains limited, and point to opportunities for expansion to other treatments and disease areas.

## Figures and Tables

**Table 1:** Patient characteristics presented as numbers, percentages, medians, and ranges.

Patient Characteristic	Included Patients	Patients with >2 Non-Responded Questionnaires
	Number (%) N = 30	Number (%) N = 6
Age; median (range)	77 years (61–87)	78 years (68–87)
Gender - Female - Male	12 (40) 18 (60)	5 (83) 1 (17)
Living status - Cohabiting - Living alone	25 (83) 5 (17)	5 (83) 1 (17)
Years since primary MM diagnosis; median (range)	6 years (1–28)	3 years (1–16)
New or ongoing on daratumumab - New - Ongoing	12 (40) 18 (60)	2 (33) 4 (67)

**Table 2:** Reason for red-coded responses.

Reason for Red Coded Response	Treatment Administered N = 30	Treatment Canceled N = 7
Rash, shortness of breath, and other side effects	1	0
Shortness of breath	3	0
Shortness of breath, fever, and diarrhea	0	1
Shortness of breath, fever, diarrhea, constipation, and other side effects	1	0
Shortness of breath and diarrhea	1	0
Shortness of breath, fever, constipation, and other side effects	0	1
Shortness of breath and other side effects	3	0
Fever	2	3
Fever and other side effects	0	1
Diarrhea	6	1
Diarrhea and constipation	1	0
Diarrhea, constipation, and other side effects	1	0
Diarrhea and other side effects	1	0
Constipation	2	0
Constipation and other side effects	1	0
Other side effects	7	0

## Data Availability

The raw data supporting the conclusions of this article are available from the corresponding authors upon reasonable request. The study was conducted in accordance with Danish data protection regulations (registration no. 21/39486).

## Abbreviations

The following abbreviations are used in this manuscript:

COREQ	Consolidated Criteria for Reporting Qualitative Research
CTCAE	Common Terminology Criteria for Adverse Events
EORTC	European Organization for Research and Treatment of Cancer
MM	Multiple Myeloma
PRO	Patient-Reported Outcome
SC	Subcutaneous
